# Dementia incidence and population-attributable fraction for dementia risk factors in Republic of Korea: a 12-year longitudinal follow-up study of a national cohort

**DOI:** 10.3389/fnagi.2023.1126587

**Published:** 2023-07-13

**Authors:** Song Hwangbo, Jin Young Lee, Gyule Han, Min Young Chun, Hyemin Jang, Sang Won Seo, Duk L. Na, Sungho Won, Hee Jin Kim, Dong Hui Lim

**Affiliations:** ^1^Department of Neurology, Samsung Medical Center, Sungkyunkwan University School of Medicine, Seoul, Republic of Korea; ^2^Alzheimer’s Disease Convergence Research Center, Samsung Medical Center, Seoul, Republic of Korea; ^3^Department of Statistics, Chung-Ang University, Seoul, Republic of Korea; ^4^Department of Ophthalmology, Samsung Medical Center, Sungkyunkwan University School of Medicine, Seoul, Republic of Korea; ^5^Department of Neurology, Yongin Severance Hospital, Yonsei University Health System, Yongin-si, Republic of Korea; ^6^Department of Health Sciences and Technology, SAIHST, Sungkyunkwan University, Seoul, Republic of Korea; ^7^Department of Public Health Sciences, Seoul National University, Seoul, Republic of Korea; ^8^Samsung Advanced Institute for Health Sciences and Technology (SAIHST), Sungkyunkwan University, Seoul, Republic of Korea

**Keywords:** dementia, Alzheimer’s disease dementia, national cohort, risk factor, population-attributable fraction

## Abstract

**Background:**

We aimed to investigate the incidence of dementia by age and year as well as the population-attributable fractions (PAFs) for known dementia risk factors in Republic of Korea.

**Methods:**

A 12-year, nationwide, population-based, retrospective cohort study was conducted. We used customized health information from the National Health Insurance Service (NHIS) data from 2002 to 2017. We analyzed age- and sex-adjusted incidence rates and PAF of dementia for each risk factor such as depression, diabetes, hemorrhagic stroke, ischemic stroke, hypertension, osteoporosis and physical inactivity using Levin’s formula.

**Results:**

Of the 794,448 subjects in the dementia-free cohort, 49,524 (6.2%) developed dementia. Dementia incidence showed annual growth from 1.56 per 1,000 person-years in 2006 to 6.94 per 1,000 person-years in 2017. Of all dementia cases, 34,544 subjects (69.8%) were female and 2,479 subjects (5.0%) were early onset dementia. AD dementia accounted for 66.5% of the total dementia incidence. Considering relative risk and prevalence, physical inactivity attributed the greatest to dementia (PAF, 8.1%), followed by diabetes (PAF, 4.2%), and hypertension (PAF, 2.9%). Altogether, the significant risk factors increased the risk of dementia by 18.0% (overall PAF).

**Conclusion:**

We provided the incidence of dementia and PAFs for dementia risk factors in Republic of Korea using a 12-year, nationwide cohort. Encouraging lifestyle modifications and more aggressive control of risk factors may effectively prevent dementia.

## Introduction

As the world’s population ages, the number of dementia patients gradually increases along with increased medical and societal costs for dementia care (Livingston et al., 2017). Alzheimer’s disease (AD) is the most common type of dementia that currently has no treatment for cure (Fiest et al., 2016). Therefore, identifying the incidence rate of dementia and finding effective strategies to prevent dementia is important in planning for public healthcare policies.

The prevalence and incidence of dementia vary across countries. According to the World Health Organization, approximately 47 million people worldwide suffer from dementia. In the United States, the prevalence of AD and related dementia in people over the age of 65 years was reported to be 11.5% (Matthews et al., 2019). In the Framingham Heart Study, the incidence of AD dementia and vascular dementia in those over the age of 60 was 2.0 per 100 persons over 5 years (Satizabal et al., 2016). In the English population, the incidence of AD dementia in those over the age of 65 was 7.06 per 1,000 person-years (Cadar et al., 2018). In the Dutch population, the incidence of AD dementia in people over the age of 60 was 5.77 per 1,000 person-years (van Bussel et al., 2017). In Japan, the incidence of all-cause dementia and AD dementia in those over 65 years of age was 41.6 and 28.2 per 1,000 person-years (Ohara et al., 2017; Table 1). In the South Korean population, the prevalence of dementia was gradually increasing, and the prevalence in people over the age of 65 was reported to be approximately 10.16% (Korean Dementia Observatory 2019) (Cho et al., 2014). However, there is no detailed data on dementia incidence in the South Korean population.

**TABLE 1:** Trends in dementia incidence in western and eastern high-income countries.

Study (reference)	Country	Design	The incidence of dementia	Years of Assessment
Framingham heart study (Satizabal et al., 2016)	United States	Prospective cohort study	AD dementia and vascular dementia: 2.0 per 100 person over 5 years	Late 2000s–early 2010s
English longitudinal study of Ageing (Cadar et al., 2018)	England	Prospective cohort study	AD dementia: 7.06 per 1,000 person-year	2002–2015
Dementia incidence trend over 1992–2014 in Netherlands (van Bussel et al., 2017)	Netherlands	Prospective cohort study	AD dementia: 5.77 per 1,000 person-year	1992–2014
Hisayama study (Ohara et al., 2017)	Japan	Prospective cohort study	All-cause dementia: 41.6 per 1,000 person-year AD dementia: 25.2 per 1,000 person-year	2000–2012

AD, Alzheimer’s disease.

There are various known risk factors for dementia, and these factors have varying degrees of impact on dementia. Although some risk factors, including age, sex, race, familial history and genetic factors, are not modifiable, there are several modifiable risk factors that can be acted upon (Livingston et al., 2020; Qiu et al., 2022). A recent study suggested that modifiable risk factors, such as less education, hearing loss, traumatic brain injury, hypertension, alcohol misuse, obesity, smoking, depression, social isolation, physical inactivity, diabetes, and air pollution, contribute to around 39% of dementia (Livingston et al., 2020). Because no medication is known to cure dementia, current trends in the management of dementia focus on its prevention (Livingston et al., 2017). Risk factors that have higher prevalence and higher risk for dementia would contribute more to dementia incidence. Thus, when establishing population-level public health strategies for dementia prevention, the prevalence and relative risk of each risk factor in the target population should be considered.

Using National Health Insurance Service data (NHIS), we first aimed to provide dementia incidence by age and year in each sex in Republic of Korea. Second, we evaluated the relative risk of each risk factor for dementia and estimated the attributable fraction for dementia in Republic of Korea. We used customized health information from the National Health Insurance Service (NHIS) data from 2002 to 2017.

## Materials and methods

This study was performed in accordance with the principles of the Declaration of Helsinki and was approved by the Samsung Medical Center Institutional Review Board (SMC IRB 2018-08-017). The requirement for informed consent from all subjects was waived as we used the national cohort data.

### Data source

For this national cohort study, we used customized health information from the National Health Insurance Service (NHIS) data, which includes more than 99% of Koreans (approximately 50 million).^1^ The NHIS operates national health check-up, and the NHIS database includes personal information, health insurance claim codes (procedures and prescriptions), diagnostic codes from the Korean Standard Classification of Diseases, 7th Revision (KCD-7), which is based on the International Classification of Diseases, 10th Revision (ICD-10), socioeconomic data (residence and income), and health examination data for each participant from 2002 to 2017. Data on body mass index (BMI), and behavioral characteristics, such as smoking status, alcohol consumption, and physical activity, were merged from the general health examinations in the NHIS database which were assessed at baseline (2006). The behavioral characteristics were assessed via standardized questionnaires.

### Definition of dementia

Dementia was defined by both ICD-10 diagnosis codes (F00, F01, F02, F03, G30, or G31) and medication prescriptions with donepezil, galantamine, rivastigmine, or memantine. Dementia was subdivided into AD dementia with ICD-10 codes F00 and G30.

### Dementia-free cohort

**FIGURE 1:**
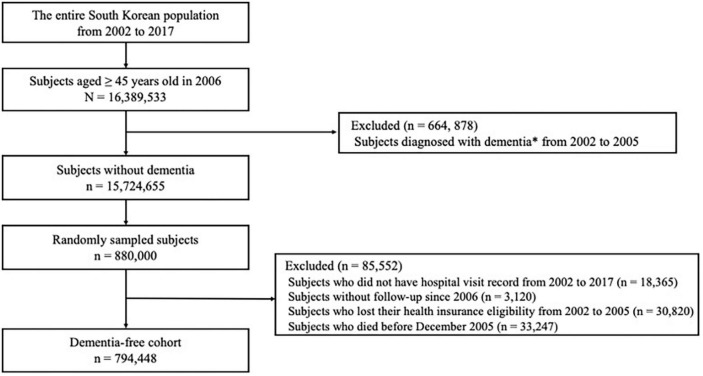
Flow chart of eligible subjects in the dementia-free cohort. *Diagnosis of dementia was based on both International Classification of Diseases-10 diagnosis codes (F00, F01, F02, F03, G30, or G31) and medication prescriptions (donepezil, galantamine, rivastigmine, or memantine).

In the NHIS database, a total of 16,389,533 subjects aged 45 years or older were identified in 2006. We excluded 664,878 subjects diagnosed with dementia between January 1, 2002, and December 31, 2005 and identified a total of 15,724,655 subjects without dementia. Then, to meet the size limit for data extraction, we selected 880,000 subjects using simple random sampling method. We additionally excluded subjects who did not have hospital visit records from 2002 to 2017 (18,365 subjects), subjects without follow-up since 2006 (3,120 subjects), subjects who lost their health insurance eligibility from 2002 to 2005 (30,820 subjects), and subjects who died before December 2005 (33,247 subjects). Finally, 794,448 subjects were followed up from 2006 to 2017 (Figure 1).

### Risk factors and covariates

With respect to risk factors for dementia, we considered coronary heart disease, depression, diabetes, hemorrhagic stroke, ischemic stroke, hypertension, hyperlipidemia, osteoporosis, physical inactivity, smoking status, heavy alcohol consumption, household income, and BMI. Age and sex were considered as covariates. Among these risk factors, coronary heart disease, depression, diabetes, hemorrhagic stroke, ischemic stroke, hypertension, hyperlipidemia, and osteoporosis were diagnosed using the corresponding ICD-10 codes at baseline. BMI was categorized into three groups: normal (18.5–22.9 kg/m^2^), underweight (<18.5 kg/m^2^), overweight (23–24.9 kg/m^2^), or obesity (≥25 kg/m^2^) (Seo et al., 2019). Smoking was defined as ever-smoker, indicating person who has smoked at least one hundred cigarettes and cigars in lifetime. Physical inactivity was defined as the absence of moderate- or high-intensity physical activity. Moderate- or high-intensity physical activity was defined as having physical activity for more than 10 min at least once a week. Heavy alcohol consumption was defined as ≥30 g of alcohol consumption per day. Low socioeconomic status was defined as lower 20% of household income.

### Statistical analysis

Our cohort was dementia free at baseline and was followed up for dementia incidence. We calculated the crude, age-specific, and sex-specific dementia incidence rates. CIs of incidence rates were obtained under the assumption that the number of events follows a Poisson distribution. Age- and sex-adjusted incidence rates were summed to obtain the standardized Korean incidence rates by weighting them with their proportions in the general Korean population. Furthermore, we calculated the relative risks (RRs) using log-binomial regression to adjust for the effect of covariates (McNutt et al., 2003). The response variables were assumed to follow a binomial distribution, and a logarithm was used as a link function. Univariable and multivariable log-binomial models were adopted to assess the risk factors associated with dementia so that the RR of each risk factor could be calculated. The multivariable model included factors that showed statistical significance in the univariate analysis (Supplementary Table 1). The model was adjusted for age, sex, BMI, smoking status, alcohol consumption, socioeconomic status, and physical activity. We also calculated the population-attributable fraction (PAF) using Levin’s formula:


$$P\,A\,F=\frac{P_{r\,i\,s\,k}\times \left(R\,R-1\right)}{1+P_{r\,i\,s\,k}\times \left(R\,R-1\right)}.$$

In our PAF analyses we used a nationwide representative cohort data and RRs were estimated after adjusting the effect of other important covariates by using a generalized linear model (GLM) with a log link function for binomial data. Our GLM analyses included several covariates, and correlations between RR were adjusted. Therefore, we did not need to adjust the communality.

Estimates using the log-binomial model after adjusting for covariates were utilized for RR. The Health Insurance Review & Assessment Service-National Patient Sample (HIRA-NPS) and the Korea National Health and Nutrition Examination Survey data, a representative sample database of the Korean population, were used to investigate the prevalence of risk factors. The prevalence of coronary heart disease, depression, diabetes, hemorrhagic stroke, ischemic stroke, hypertension, hyperlipidemia, and osteoporosis for those aged 45 years and older were estimated using the HIRA-NPS dataset (2018). The prevalence of underweight, overweight, obesity, physical inactivity, smoking, heavy alcohol consumption, and low socioeconomic status among those aged 45 years and older were estimated by the Korea National Health and Nutrition Examination Survey (2018).


$$O\,v\,e\,r\,a\,l\,l\,P\,A\,F=1-\left(1-P\,A\,F_{1}\right)\times \left(1-P\,A\,F_{2}\right)\times \left(1-P\,A\,F_{3}\right)\times \ldots$$

All statistical analyses were performed using SAS (version 9.3; SAS Institute, Inc., Cary, NC, USA), R Statistical Software (version 3.5; Foundation for Statistical Computing, Vienna, Austria), and Rex (Version 3.0.3, RexSoft Inc., Seoul, Republic of Korea). The significance level for the statistical analyses was set at 0.05.

## Results

Among the 794,448 subjects in the dementia-free cohort, 364,765 (45.9%) were males, and 429,683 subjects (54.1%) were females. The most prevalent risk factors at baseline were physical inactivity (56.9%), followed by hypertension (32.4%), smoking (30.9%), and diabetes (21.3%) (Table 2). Among 794,448 subjects in the dementia-free cohort, 49,524 subjects developed all-cause dementia between 2006 and 2017. Compared to subjects who were dementia-free at follow-up, subjects who developed dementia had a female predominance and had a higher prevalence of hypertension, diabetes, dyslipidemia, coronary heart disease, ischemic stroke, hemorrhagic stroke, depression, osteoporosis, underweight, physical inactivity, and low socioeconomic status.

**TABLE 2:** Demographics of study subjects.

Characteristics at initial visit	Total (*n* = 794,448)	Dementia-free at follow-up (*n* = 744,924)	Incident dementia at follow-up (*n* = 49,524)
**Sex**			
Male (%)	364,765	349,785 (47.0%)	14,980 (30.2%)
Female (%)	429,683	395,139 (53.0%)	34,544 (69.8%)
**Age, year**			
**Age groups, year**			
45–50	219,972	219,956 (29.5%)	16 (0.03%)
50–55	161,729	161,517 (21.7%)	212 (0.4%)
55–60	114,461	113,702 (15.3%)	759 (1.5%)
60–65	91,648	90,156 (12.1%)	1,492 (3.0%)
65–70	79,279	75,859 (10.2%)	3,420 (6.9%)
70–75	56,999	49,522 (6.6%)	7,477 (15.1%)
75–80	35,753	24,036 (3.2%)	11,717 (23.7%)
80–85	21,063	8,881 (1.2%)	12,182 (24.6%)
85 +	13,544	1,295 (0.2%)	12,249 (24.7%)
Hypertension (%)	257,273	229,407 (30.8%)	27,866 (56.3%)
Diabetes mellitus (%)	169,092	151,559 (20.3%)	17,533 (35.4%)
Dyslipidemia (%)	147,923	135,347 (18.2%)	12,576 (25.4%)
Coronary heart disease (%)	92,466	82,527 (11.1%)	9,939 (20.1%)
Ischemic stroke (%)	33,178	27,550 (3.7%)	5,628 (11.4%)
Hemorrhagic stroke (%)	4,419	3,802 (0.5%)	617 (1.2%)
Depression (%)	64,530	57,481 (7.7%)	7,049 (14.2%)
Osteoporosis (%)	114,386	100,467 (13.5%)	13,919 (28.1%)
**BMI^a,b^, kg/m^2^**			
Underweight (%)	7,891	7,099 (2.2%)	792 (4.3%)
Normal (%)	216,137	204,550 (62.3%)	11,587 (62.5%)
Overweight (%)	94,257	89,523 (27.3%)	4,734 (25.5%)
Obesity (%)	122,811	116,657 (35.5%)	6,154 (33.2%)
Physical inactivity^a^ (%)	197,448	184,558 (56.2%)	12,890 (69.6%)
Heavy alcohol consumption^a^ (%)	28,176	27,252 (8.3%)	924 (4.99%)
Smoking^a^ (%)	107,164	103,204 (31.4%)	3,960 (21.4%)
Low socioeconomic status	47,272	44,177 (13.5%)	3,095 (16.7%)

BMI, body mass index.

^a^Total *N* = 346,839; dementia-free *n* = 328,306; dementia *n* = 18,533.

^b^BMI was classified as normal (18.5–22.9 kg/m^2^), underweight (<18.5 kg/m^2^), overweight (23–24.5 kg/m^2^), and obese (≥25 kg/m^2^).

### Dementia incidence by year, age, and sex

**FIGURE 2:**
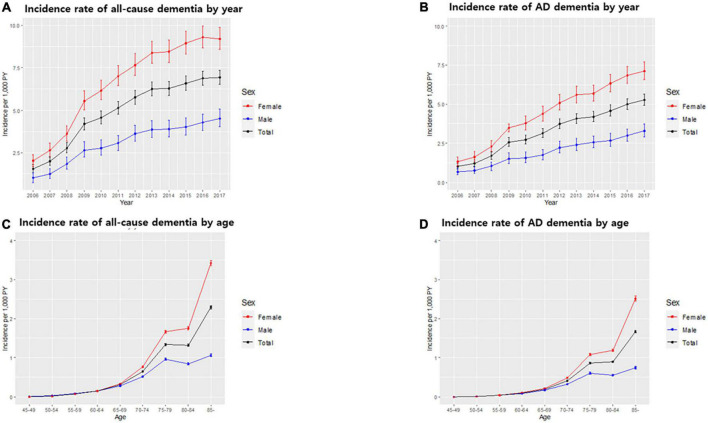
Incidence rate of dementia in the South Korean population. Incidence rate of all-cause dementia **(A)** and Alzheimer’s disease (AD) dementia **(B)** by year. Incidence rate of all-cause dementia **(C)** and AD dementia **(D)** by age. Bars represent 95% confidence intervals.

Of all patients with dementia, 66.5% (32,949 subjects) had AD dementia. The incidence rate of all-cause dementia showed annual growth, increasing from 1.56 per 1,000 person-years in 2006 to 6.94 per 1,000 person-years in 2017. The incidence rate of AD dementia also showed annual growth, increasing from 1.01 per 1,000 person-years in 2006 to 5.27 per 1,000 person-years in 2017 (Table 4 and Figures 2A, B).

The incidence of dementia increases with age. In our cohort, among 49,524 subjects who developed dementia, 2,479 subjects (5.0%) were early onset who were diagnosed with dementia younger than 65 years of age. The incidence rate of early onset dementia (EOD) was 0.24 per 1,000 person-years, while the incidence rate of late-onset dementia was 5.91 per 1,000 person-years. Among dementia subtypes, the incidence rate of early onset AD (EOAD) was 0.14 per 1,000 person-years, while the incidence rate of late-onset AD was 4.01 per 1,000 person-years (Table 3 and Figures 2C, D).

**TABLE 3:** Dementia incidence by year.

	No. of person-years			No. of new all-cause dementia cases (crude)			All-cause dementia incidence rate^a^ (95% CI) (per 1,000 person-years)			No. of new AD dementia cases (crude)			AD dementia incidence rate^a^ (95% CI) (per 1,000 person-years)		
	**Total**	**Male**	**Female**	**Total**	**Male**	**Female**	**Total**	**Male**	**Female**	**Total**	**Male**	**Female**	**Total**	**Male**	**Female**
2006	786211.7	360728.7	425483	1150	345	805	1.56 (1.34–1.81)	1.02 (0.77–1.34)	2.03 (1.71–2.41)	744	225	519	1.01 (0.84–1.20)	0.67 (0.47–0.92)	1.31 (1.05–1.61)
2007	775327.3	355242.5	420084.9	1517	436	1081	1.99 (1.75–2.26)	1.25 (0.97–1.59)	2.65 (2.27–3.08)	922	261	661	1.21 (1.02–1.42)	0.75 (0.55–1.00)	1.62 (1.33–1.96)
2008	766358.5	350678.4	415680	2178	672	1506	2.78 (2.51–3.09)	1.85 (1.52–2.25)	3.61 (3.19–4.09)	1335	376	959	1.70 (1.49–1.95)	1.04 (0.74–1.29)	2.3 (1.97–2.68)
2009	752669	344015	408654	3342	988	2354	4.19 (3.84–4.57)	2.66 (2.24–3.15)	5.54 (5.02–6.13)	2031	557	1474	2.55 (2.28–2.85)	1.51 (1.20–1.88)	3.47 (3.23–3.74)
2010	740455.7	338153.1	402302.6	3710	1053	2657	4.56 (4.19–4.96)	2.77 (2.34–3.27)	6.16 (5.61–6.78)	2223	595	1628	2.73 (2.46–3.03)	1.56 (1.26–1.94)	3.77 (3.36–4.24)
2011	727637.3	332125.2	395512	4257	1206	3051	5.13 (4.76–5.52)	3.06 (2.65–3.53)	7.00 (6.42–7.62)	2588	683	1905	3.13 (2.85–3.43)	1.74 (1.43–2.10)	4.38 (3.93–4.87)
2012	715996.8	326566.8	389430	4845	1457	3388	5.75 (5.35–6.15)	3.61 (3.16–4.11)	7.66 (7.06–8.31)	3139	896	2243	3.73 (3.42–4.06)	2.22 (1.88–2.62)	5.09 (4.61–5.62)
2013	699879.7	318984.3	380895.4	5323	1598	3725	6.26 (5.83–6.65)	3.87 (3.40–4.38)	8.38 (7.76–9.04)	3462	991	2471	4.07 (3.76–4.41)	2.41 (2.05–2.81)	5.59 (5.09–6.13)
2014	684968.9	311745.3	373223.6	5445	1652	3793	6.27 (5.87–6.69)	3.90 (3.43–4.42)	8.45 (7.83–9.11)	3627	1085	2542	4.19 (3.88–4.54)	2.57 (2.20–2.98)	5.69 (5.20–6.23)
2015	668607.2	303636.9	364970.3	5757	1733	4024	6.58 (6.16–7.02)	4.01 (3.54–4.55)	8.94 (8.30–9.63)	4000	1154	2846	4.58 (4.24–4.95)	2.68 (2.30–3.12)	6.33 (5.80–6.91)
2016	649304.6	293385.7	355918.9	6167	1930	4237	6.88 (6.48–7.29)	4.28 (3.83–4.77)	9.30 (8.68–9.95)	4465	1344	3121	4.99 (4.66–5.34)	2.99 (2.61–3.40)	6.85 (6.33–7.42)
2017	569072.2	252403.8	316668.3	5833	1910	3923	6.94 (6.53–7.36)	4.52 (4.04–5.05)	9.20 (8.58–9.87)	4413	1390	3023	5.27 (4.92–5.64)	3.29 (2.89–3.74)	7.12 (6.57–7.70)

CI, confidence interval; AD, Alzheimer’s disease.

^a^Adjusted for age and sex.

With respect to dementia incidence by sex, of all dementia patients, more females (34,544; 69.8%) than males (14,980; 30.2%) developed dementia. The incidence rates of all-cause dementia and AD dementia in males were lower than those in females over the total follow-up period (Table 3). There was no significant difference in the incidence rate by age between males and females aged <65 years. However, in the age group over 65 years, the incidence rate of all-cause dementia and AD dementia by age in females was higher than that in males, and the gap of the incidence rate between males and females increased with age (Table 3 and Figures 2C, D). The incidence rate of all-cause EOD was 0.27 per 1,000 person-years in males and 0.24 per 1,000 person-years in females. Among dementia subtypes, the incidence rate of EOAD was 0.13 per 1,000 person-years in males and 0.15 per 1,000 person-years in females.

### Risk factors for all-cause dementia and AD dementia

In the multivariable log-binomial regression model, depression (RR 1.25, 95% CI 1.21–1.30), diabetes (RR 1.19, 95% CI 1.16–1.22), ischemic stroke (RR 1.19, 95% CI 1.14–1.23), physical inactivity (RR 1.12, 95% CI 1.09–1.15), osteoporosis (RR 1.10, 95% CI 1.07–1.13), and hypertension (RR 1.08, 95% CI 1.04–1.11) increased risk of all-cause dementia (Table 4). In the same analysis depression (RR 1.29, 95% CI 1.24–1.35), hemorrhagic stroke (RR 1.22, 95% CI 1.05–1.42), diabetes (RR 1.18, 95% CI 1.14–1.22), physical inactivity (RR 1.12, 95% CI 1.08–1.16), ischemic stroke (RR 1.12, 95% CI 1.07–1.19), osteoporosis (RR 1.08, 95% CI 1.04–1.12), and hypertension (RR 1.06, 95% CI 1.02–1.10) increased the risk of AD dementia (Table 5).

**TABLE 4:** Dementia incidence by age.

	No. of person-years			No. of new all-cause dementia cases (crude)			All-cause dementia incidence rate^a^ (95% CI) (per 1,000 person-years)			No. of new AD dementia cases (crude)			AD dementia incidence rate^a^ (95% CI) (per 1,000 person-years)		
	**Total**	**Male**	**Female**	**Total**	**Male**	**Female**	**Total**	**Male**	**Female**	**Total**	**Male**	**Female**	**Total**	**Male**	**Female**
All age	8558143	3897537	4660606	49524	14980	34544	6.16 (6.03–6.30)	3.92 (3.76–4.09)	8.16 (7.97–8.37)	32949	9557	23392	4.17 (4.06–4.28)	2.54 (2.41–2.67)	5.62 (5.46–5.79)
45–49	680317.7	332120.7	348197	16	11	5	0.00 (0.00–0.01)	0.01 (0.00–0.01)	0.00 (0.00–0.01)	7	5	2	0.00 (0.00–0.00)	0.00 (0.00–0.01)	0.00 (0.00–0.00)
50–54	1577858	768229.6	809628.4	212	116	96	0.02 (0.02–0.03)	0.03 (0.02–0.03)	0.02 (0.02–0.02)	107	59	48	0.01 (0.01–0.01)	0.01 (0.01–0.02)	0.01 (0.01–0.01)
55–59	1867539	897487.3	970052	759	371	388	0.07 (0.07–0.08)	0.08 (0.07–0.09)	0.07 (0.06–0.08)	433	192	241	0.04 (0.04–0.05)	0.04 (0.04–0.05)	0.04 (0.04–0.05)
60–64	1391240	658621.9	732617.7	1492	691	801	0.15 (0.14–0.16)	0.15 (0.14–0.16)	0.15 (0.14–0.16)	903	384	519	0.09 (0.09–0.10)	0.08 (0.08–0.09)	0.10 (0.09–0.11)
65–69	1095942	500143.1	595798.9	3420	1430	1990	0.31 (0.30–0.32)	0.28 (0.27–0.30)	0.33 (0.31–0.34)	2161	883	1278	0.19 (0.19–0.20)	0.17 (0.16–0.19)	0.21 (0.20–0.22)
70–74	854618.7	364243.6	490375.1	7477	2671	4806	0.65 (0.64–0.67)	0.52 (0.50–0.54)	0.76 (0.74–0.78)	4693	1640	3053	0.41 (0.40–0.42)	0.32 (0.31–0.34)	0.48 (0.47–0.50)
75–79	579088.9	221986	357103	11717	3700	8017	1.34 (1.31–1.36)	0.96 (0.93–0.99)	1.66 (1.62–1.70)	7549	2333	5216	0.86 (0.84–0.88)	0.60 (0.58–0.63)	1.08 (1.05–1.11)
80–84	370403.4	118762.1	251641.2	12182	3320	8862	1.32 (1.30–1.35)	0.84 (0.81–0.87)	1.75 (1.71–1.79)	8205	2191	6014	0.89 (0.87–0.91)	0.55 (0.53–0.58)	1.19 (1.16–1.22)
85–	141135.4	35942.62	105192.8	12249	2670	9579	2.29 (2.25–2.33)	1.06 (1.02–1.10)	3.42 (3.36–3.49)	8891	1870	7021	1.66 (1.63–1.70)	0.74 (0.71–0.78)	2.51 (2.45–2.57)

CI, confidence interval; AD, Alzheimer’s disease.

^a^Adjusted for age and sex.

**TABLE 5:** Relative risk and population-attributable fraction of risk factors for all-cause dementia and AD dementia.

	Risk factor prevalence	All-cause dementia		AD dementia	
		**RR^a^ (95% CI)**	**PAF**	**RR^a^ (95% CI)**	**PAF**
Hypertension	40.10%	1.08 (1.04–1.11)	2.9% (1.7–4.1)	1.06 (1.02–1.10)	2.3% (0.8–3.8)
Diabetes	23.20%	1.19 (1.16–1.22)	4.2% (3.5–4.9)	1.18 (1.14–1.22)	4.0% (3.1–4.9)
Depression	8.50%	1.25 (1.21–1.30)	2.1% (1.7–2.5)	1.29 (1.24–1.35)	2.4% (2.0–3.1)
Osteoporosis	12.20%	1.10 (1.07–1.13)	1.2% (0.8–1.6)	1.08 (1.04–1.12)	1.0% (0.5–1.5)
Physical inactivity	73.30%	1.12 (1.09–1.15)	8.1% (6.1–10.1)	1.12 (1.08–1.16)	8.2% (5.6–10.7)
Ischemic stroke	5.00%	1.19 (1.14–1.23)	0.9% (0.7–1.1)	1.12 (1.07–1.19)	0.6% (0.3–0.9)
Hemorrhagic stroke	0.60%	1.09 (0.97–1.23)		1.22 (1.05–1.42)	0.1% (0.0–0.2)
Hyperlipidemia	35.60%	1.02 (0.99–1.05)		1.01 (0.97–1.05)	
Coronary heart disease	8.10%	0.99 (0.96–1.02)		0.99 (0.95–1.03)	
Smoking	55.90%	1.03 (0.99–1.07)			
Obesity	37.10%	0.93 (0.90–0.96)		0.91 (0.88–0.95)	

AD, Alzheimer’s disease; RR, relative risk; CI, confidence interval; PAF, population-attributable fraction.

^a^Adjusted for sex, age, and each risk factor.

### PAF for all-cause dementia and AD dementia

Among the risk factors, physical inactivity attributed the greatest to all-cause dementia (PAF, 8.1%), followed by diabetes (PAF, 4.2%) and hypertension (PAF, 2.9%). The overall PAF for all-cause dementia was 18.0%. With respect to AD dementia, physical inactivity attributed the greatest (PAF, 8.2%) followed by diabetes (PAF, 4.0%) and depression (PAF, 2.4%). The overall PAF for AD dementia was 17.4%.

## Discussion

In this study, we identified dementia incidence and dementia risk factors using the Korean national cohort data. The incidence rate of all-cause dementia continuously increased from 2006 to 2017, reaching 6.94 (4.53 in males, 9.20 in females) per 1,000 person-years in 2017. Of the total incidence of all-cause dementia, 5.0% of cases were EOD. AD dementia accounted for 66.5% of the total dementia incidence. The incidence rate of AD dementia also increased from 2006 to 2017, reaching 5.27 (3.29 in males, 7.20 in females) per 1,000 person-years in 2017. Of several risk factors, physical inactivity and diabetes contributed the highest to the occurrence of dementia.

We identified an increasing trend in the incidence of dementia in Republic of Korea over 10 years. Trends in dementia incidence vary according to study design, population, and time of study. Declining or stable trends were reported in several western high-income countries such as the United States, the UK, Sweden, Netherlands, Canada, and France during the 2000s and the early 2010s (Manton et al., 2005; Schrijvers et al., 2012; Matthews et al., 2013, 2016; Qiu et al., 2013; Satizabal et al., 2016; Derby et al., 2017; Wu et al., 2017; Stephan et al., 2018; Livingston et al., 2020). Multiple factors appear to have a complex influence on these trends. Several previous studies suggested that higher educational level, decreasing smoking prevalence, advances in management of vascular risk factors and cardiovascular diseases, and healthier lifestyles, might explain the decrease in dementia incidence (Larson et al., 2013; de Bruijn et al., 2015; Jørgensen et al., 2015; Grasset et al., 2016; Satizabal et al., 2016; Langa et al., 2017; Livingston et al., 2017, 2020; Wu et al., 2017). Conversely, several studies showed annual growth in the incidence of dementia. In the Danish population aged 65–74 years, the incidence of dementia increased from 0.79 per 1,000 person-years in 2000 to 1.52 per 1,000 person-years in 2009 (Jørgensen et al., 2015). In the Welsh population aged 60 years and older, the incidence of AD dementia increased from 1.4 per 1,000 person-years in 1999 to 1.9 per 1,000 person-years in 2010 (Engelhardt et al., 2002). In addition, increasing trends have been reported in other far-eastern high-income countries such as Japan, Hong Kong, and Taiwan (Wu et al., 2017; Gao et al., 2019; Livingston et al., 2020). The longevity or aging population is the main cause of increasing dementia incidence. In addition, along with economic growth more screening tests for dementia are performed. In Republic of Korea, since the National Institution of Dementia was established in 2013 as a national project, the dementia screening service was initiated nationwide. Consequently, previously undiagnosed patients were diagnosed with dementia, which resulted in an increased dementia incidence.

As expected, the incidence of dementia increased with age. Of the total dementia incidence, EOD accounted for 5.0%, and the incidence rate of EOD was 0.24 per 1,000 person-years in Republic of Korea. In several community-based or nationwide studies, the frequency of EOD among all-cause dementia ranges from 7.3 to 45.3% (Fujihara et al., 2004; Yokota et al., 2005; Garre-Olmo et al., 2010; Noh et al., 2014). According to the results of two recent population-based studies, the incidence rate of EOD was 0.13 per 1000 person-years, and EOD cases were 6.9% of all-cause dementia in Spain (Garre-Olmo et al., 2010; Noh et al., 2014). In the UK, the incidence rate of EOD was 0.12 per 1000 person-years (Mercy et al., 2008; Noh et al., 2014). The incidence rate of AD dementia also increased with age. Of the total AD dementia incidence, EOAD accounted for 4.4%, and the incidence rate of EOAD was 0.14 per 1,000 person-years. According to the results of nationwide studies in several other countries, the incidence rate of EOAD varied from 0.024 to 0.226 per 1000 person-years (Vieira et al., 2013).

We observed that in the age group over 65 years, the incidence rate of all-cause dementia and AD dementia in females was significantly higher than that in males. Females accounted for 69.8% of all patients with dementia. There are several possible reasons for this discrepancy. First, there are risk factors for dementia which differ by sex in frequency and prevalence. For example, a low education level, depression, and limited social activities are more frequent in females (Rocca et al., 2014; Mielke, 2018). Second, since the life expectancy for females is longer than for males, females have greater lifetime risk for dementia. In addition, a physiologic condition such as menopause, a dramatic fall of estrogen and progesterone in late middle age, may increase dementia risk for females (Scheyer et al., 2018).

We found that the contribution of the three highest modifiable risk factors (physical inactivity, diabetes, and hypertension) to all-cause dementia was 15.2%, and the contribution of the three highest modifiable risk factors (physical inactivity, diabetes, and depression) to AD dementia was 15.8%. A Japanese cohort study conducted in 2006 showed similar results to our study, that physical inactivity, diabetes and severe psychological distress are the highest modifiable risk factors for dementia (Kotaki et al., 2019). On the other hand, our results were different from the PAFs reported by Livingston et al. (2017, 2020). In the previous study, the RR and the prevalence of each risk factor were obtained from other reports, whereas in our study, the RR and prevalence of each risk factor were obtained from the Korean national cohort, which we believe better reflects the situation of dementia in Republic of Korea. In addition, the risk factors included in the current study was different from those included in previous studies. In Republic of Korea, the most prevalent modifiable risk factors were physical inactivity (73.3%), hypertension (40.1%), and diabetes (23.2%). There is lack of physical activity in the Korean adult population (Kim et al., 2002). In addition, the prevalence of type 2 diabetes is rapidly increasing in Asian populations (Ramachandran et al., 2010; Kim et al., 2015) including South Korean population. Therefore, more aggressive monitoring and control of these risk factors will enable more effective dementia prevention. Especially, encouraging lifestyle modifications to increase physical activity is critical in dementia prevention. Ultimately, effective dementia prevention strategies may reduce the medical and societal costs of dementia management and burden on the patient’s family or guardians.

The present study has several limitations. First, since the presence of risk factors was evaluated only at baseline, newly developed risk factors during the follow-up period were not considered. Second, because data on the education level, hearing loss, traumatic brain injury, social isolation, and air pollution were not available from the NHIS data, the PAFs of these factors could not be estimated. In addition, the relationships between the risk factors are complex, as one may affect the other. For example, lack of physical activity may lead to hypertension and diabetes, ultimately increasing the risk for dementia. It will be necessary to investigate the contribution of various risk factors to dementia considering their complex relationships in future studies. Third, because dementia occurrence was defined by the first prescription of dementia medicine and diagnosis with dementia codes based on the Korean Standard Classification of Diseases, dementia subtypes other than AD might have been underdiagnosed. Finally, we did not calculate medication effects between risk factors.

Nevertheless, the strength of this study is that it is the first national cohort study in Republic of Korea to investigate dementia incidence by year, age, and sex using a dataset representing the South Korean population. Furthermore, since this study was conducted using data from one single national cohort, high communality was guaranteed. This study could contribute to better understand the current situation of dementia in various countries and predict about future trends in dementia worldwide.

## Conclusion

As the incidence of dementia in Republic of Korea is gradually increasing, the social and individual burden of dementia is also growing. It is important to understand the trend of dementia incidence and modifiable risk factors for dementia to establish optimal strategies for dementia prevention in each population.

## Data availability statement

The data analyzed in this study is subject to the following licenses/restrictions: the datasets used and/or analyzed during the current study are available from the corresponding authors (DL and HK) on reasonable request. Requests to access these datasets should be directed to DL, ldhlse@gmail.com.

## Ethics statement

The studies involving human participants were reviewed and approved by the Samsung Medical Center Institutional Review Board. Written informed consent for participation was not required for this study in accordance with the national legislation and the institutional requirements.

## Author contributions

DL and HK: study design and conduct. SH and JL: data analysis and writing the manuscript. JL, GH, and SW: statistical analysis. MC: acquisition of data. HJ, SS, and DN: interpretation of findings. All authors read and approved the final manuscript.

## Footnotes

1
http://nhiss.nhis.or.kr
